# Effects of Elevated β-Estradiol Levels on the Functional Morphology of the Testis - New Insights

**DOI:** 10.1038/srep39931

**Published:** 2017-01-03

**Authors:** Myles Leavy, Matthias Trottmann, Bernhard Liedl, Sven Reese, Christian Stief, Benjamin Freitag, John Baugh, Giulio Spagnoli, Sabine Kölle

**Affiliations:** 1 School of Medicine and Medical Science, University College Dublin (UCD), Dublin, Ireland; 2 Department of Urology, Klinikum Grosshadern, University of Munich, Germany; 3 Department of Urogenital Surgery, Clinics for Surgery Munich-Bogenhausen, Munich, Germany; 4 Institute of Veterinary Anatomy, Histology and Embryology, University of Munich, Germany; 5 Department of Biomedicine, University Hospital Basel, Switzerland

## Abstract

Elevated estradiol levels are correlated with male infertility. Causes of hyperestrogenism include diseases of the adrenal cortex, testis or medications affecting the hypothalamus-pituitary-gonadal axis. The aim of our study was to elucidate the effects of estradiol treatment on testicular cellular morphology and function, with reference to the treatment regimen received. Testes samples (n = 9) were obtained post-orchiectomy from male-to-female transsexuals within the age range of 26–52 years. Each patient had a minimum of 1–6 years estradiol treatment. For comparison, additional samples were obtained from microscopically unaltered testicular tissue surrounding tumors (n = 7). The tissues obtained were investigated by stereomicroscopy, histochemistry, scanning electron microscopy (SEM) and immunohistochemistry. Our studies revealed that estradiol treatment significantly decreased the diameter of the seminiferous tubules (p < 0.05) and induced fatty degeneration in the surrounding connective tissue. An increase in collagen fiber synthesis in the extracellular matrix (ECM) surrounding the seminiferous tubules was also induced. Spermatogenesis was impaired resulting in mainly spermatogonia being present. Sertoli cells revealed diminished expression of estrogen receptor alpha (ERα). Both Sertoli and Leydig cells showed morphological alterations and glycoprotein accumulations. These results demonstrate that increased estradiol levels drastically impact the human testis.

Estrogens play an important role in ensuring proper function of the male reproductive tract. In males, the main biologically active estrogen is estradiol. The primary source of this hormone is from the aromatization of testosterone^1^. Estrogen receptors alpha and beta (ERα and ERβ), together with the membrane associated G-protein-coupled functional ER (GPER), and the enzyme cytochrome P450 aromatase, which converts testosterone to estrogen, are abundant within the testis^2^,^3^,^4^. Estradiol is produced in immature germ cells, spermatozoa, in the epithelium of the efferent ductules and the proximal epididymal duct and in the Leydig cells and Sertoli cells^5^,^6^,^7^,^8^,^9^. Thus, it is possible that estradiol concentrations within the testis and semen can even exceed those found in the female vasculature^10^,^11^,^12^,^13^,^14^,^15^. The function of estradiol varies depending on the cells in which it is being produced. In testicular cells, estradiol is known to regulate numerous aspects of spermatogenesis, including proliferation, differentiation, survival and apoptosis of germ cells^16^,^17^,^18^,^19^,^20^. Estradiol is involved in the modulation of cell communication via the tight junctions of Sertoli cells and in the regulation of Leydig cell function^21^,^22^,^23^,^24^,^25^. Estradiol has been shown to play a role in proliferation, regulation of ion transport and regulation of apoptosis in Sertoli cells^26^,^27^,^28^. In addition, estradiol is responsible for the inhibition of Leydig cell testosterone production and the blocking of Leydig cell regeneration^29^,^30^. In the efferent ductules, estradiol is involved in the reabsorption of fluids, thus, affecting sperm concentration, motility and morphology^31^. As the regulation of testicular cells by estradiol shows both inhibitory and stimulatory influences, this points to a delicate, dose-dependent and temporally sensitive modulation^32^.

Estradiol’s physiological importance in the male reproductive tract has been well documented in the literature both in humans and in animal model systems. However, it is mainly in animal models that the effects of elevated estradiol levels in males has been evaluated. Previous studies have demonstrated that estradiol significantly enhanced mouse sperm fertilizing capacity *in vitro*^33^. Along with an enhanced fertilizing capacity, studies found estradiol induced the sperm acrosome reaction and capacitation in uncapacitated sperm^33^. In an *in vivo* mouse model, estradiol exposure resulted in premature capacitation of the cauda epididymal sperm, which could be correlated with decreased sperm viability in the reproductive tract of the female^34^. Chronic exposure to estradiol 3-benzoate in rats has been reported to cause significant effects on the epididymis, testis, seminal vesicle and prostate^35^. The effects reported in this study included a decrease in weight of the sex organs, impaired spermatogenesis and a reduced number of germ cells secondary to an increase in germ cell apoptosis^35^. All these observations were attributed to the negative effect of estradiol on the expression of androgen receptor (AR)^35^. Estrogen treatment has been shown to cause an increase in ERα expression and decrease in AR expression in the rat testis^35^,^36^.

Studies conducted in the late 1970 s and 1980 s have previously assessed the effects of estradiol treatment on morphology of the human testis in male-to-female transsexuals. One study showed that estradiol treatment resulted in decreased testicular size and reduced germ cell numbers^37^. Another found total absence of spermatogenic activity in the majority of patients treated with estrogens^38^. Ultrastructural studies showed a loss of the apico-basal differentiation in Sertoli cells and dedifferentiation of Leydig cells after estrogen treatment^39^,^40^. However, detailed histomorphological and histochemical analyses of the effects of long-term treatment with estradiol, specifically linked to a patients‘ treatment plans are still lacking, as stated in the literature^41^.

The purpose of this study was therefore to assess the effects of estradiol treatment on the functional morphology of the testis and on spermatogenesis in male-to-female transsexuals, with special focus on the impact of different treatment regimens on testicular alterations. This knowledge is a pivotal prerequisite to create novel therapeutic strategies for treatment of patients with hormonal disorders to restore testicular function and fertility.

## Results

### Effect of hormonal treatment on seminiferous tubule diameter

**Figure 1:**
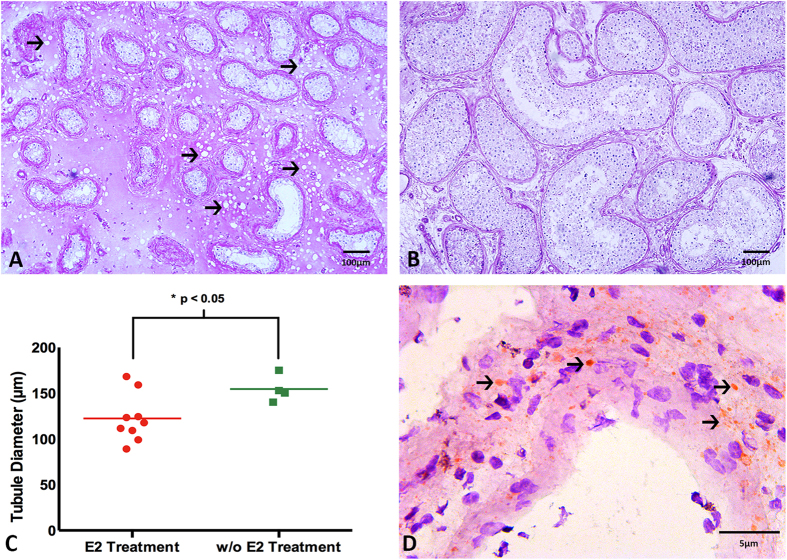
Effect of estradiol treatment on the microarchitecture of the testis. (**A**) Estradiol treated patients showed fatty degeneration within the surrounding connective tissue (→, PAS). (**B**) In comparison, the patients w/o estradiol treatment showed no vacuoles in their connective tissue (PAS). (**C**) Estradiol significantly decreased the diameter of the seminiferous tubules of the patients. Data are presented as an average of seminiferous tubule diameter (μm) of the patients with estradiol treatment (red) and w/o estradiol treatment (green). (*p < 0.05, 2-sided *t*-test). The decrease in diameter of the seminiferous tubules is seen when comparing Fig. 1A,B. (**D**) Sudan staining confirmed that the vacuoles seen in the estradiol treated patients contain fat (→).

Estradiol treatment significantly decreased the diameter of the seminiferous tubules (Fig. 1A, E2 treatment; Fig. 1B, w/o E2 treatment; Fig. 1C, p < 0.05, 2-sided *t*-test). The seminiferous tubules of patients treated with estradiol were, on average, 21% smaller in diameter than the patients without estradiol treatment (Fig. 1A,B). Following estradiol treatment, the diameter of the seminiferous tubules ranged between 89 and 175 μm compared to 148–180 μm with no treatment.

### Effect of hormonal treatment on the metabolism of the surrounding connective tissue of the testis

Following estradiol treatment, 8 out of 9 patients showed a large number of vacuoles outside the seminiferous tubules within the surrounding connective tissue (Fig. 1A, arrows). Only one patient taking estradiol medication in combination with the progestin dydrogesterone (Duphaston^®^) did not show vacuolation. Sudan Red staining confirmed that these vacuoles were fat droplets (Fig. 1D, arrows).

### Effect of hormonal treatment on the composition of the extracellular matrix (ECM) of the testis

**Figure 2:**
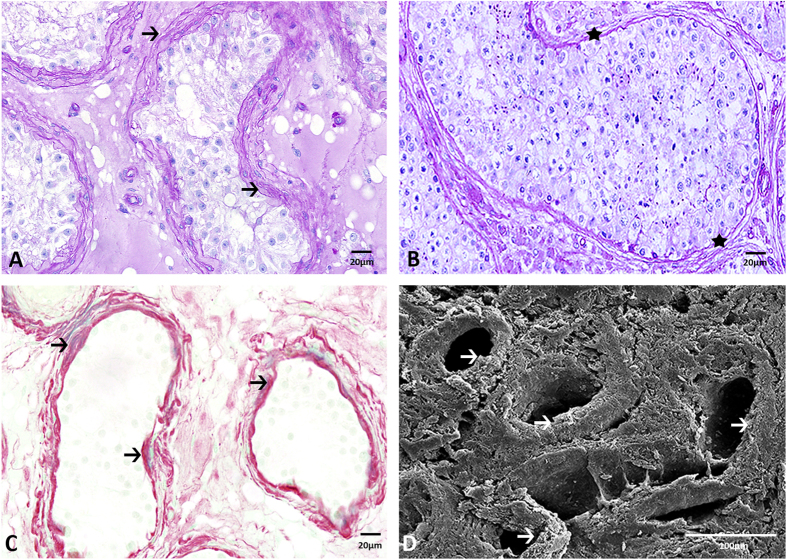
Effect of treatment with estradiol on collagen synthesis in the testis. (**A,B**) Estradiol treatment caused an increase in thickness of the seminiferous tubule ECM (**A**, →, PAS) when compared to the patient tissue w/o estradiol treatment (**B**, ★, PAS). (**C**) The red staining around the seminiferous tubules indicates the increased amount of collagen fibers after estradiol treatment (Picrosirius red stain, →). (**D**) SEM imaging confirms the increased amount of collagen fibers in the ECM of the seminiferous tubules of the estradiol treated patients (→).

After treatment, 7 of the 9 patients displayed an increased synthesis of ECM around the seminiferous tubules (Fig. 2A, arrows), as compared to the patients without estradiol treatment (Fig. 2B, stars). The ECM of the other 2 treated patients, one who received estradiol with antiandrogens over a period of 6 years and one who received estradiol only, were similar to the patients without estradiol treatment. Picrosirius red stain demonstrated that the increased thickness of the ECM was due to increased synthesis of collagen fibers (Fig. 2C, arrows). SEM confirmed the increased number of collagen fibers surrounding the tubules in the estradiol treated patients (Fig. 2D, arrows).

### Effect of hormonal treatment on spermatogenesis

**Figure 3:**
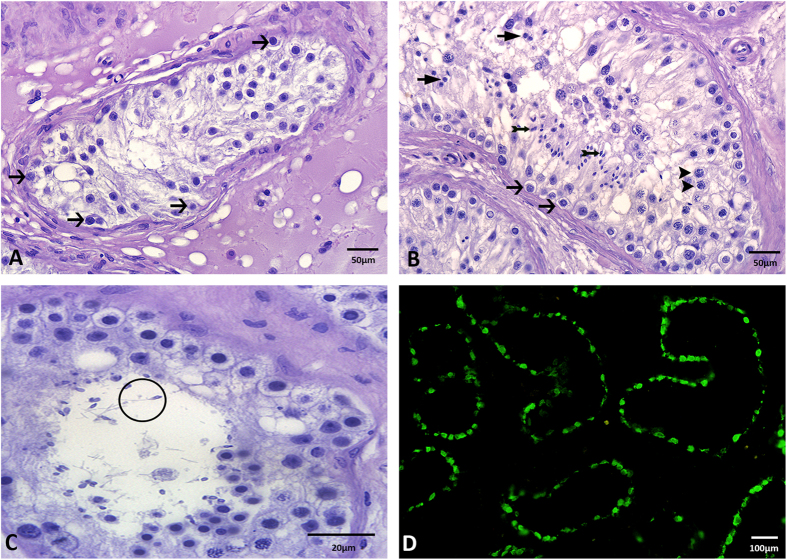
Effect of treatment with estradiol on spermatogenesis. (**A**) Estradiol treatment results in only spermatogonia being present in the seminiferous tubules (→ spermatogonia, H&E). (**B**) Seminiferous tubule from a patient w/o estradiol treatment demonstrating all of the cells of spermatogenesis (→ spermatogonia; ➤ spermatocytes; ➛ round spermatids; ➼ elongated spermatids, H&E). (**C**) Two patients receiving estradiol only or estradiol and antiandrogens for 6 years, showed single spermatozoa in the seminiferous tubules (circle, H&E). (**D**) Immunofluorescent localization of MAGEA4 in estradiol treated patients demonstrated that nearly all cells in the seminiferous tubules are spermatogonia.

Following estradiol treatment, 7 of the 9 patients showed a severe impairment in spermatogenesis (Fig. 3A) with no sperm being present in the seminiferous tubules. In comparison, the seminiferous tubules of patients without estradiol treatment contained spermatogonia (Fig. 3B, arrows), spermatocytes (Fig. 3B, arrowheads), round spermatids (Fig. 3B, arrows with compact arrowhead), and elongated spermatids (Fig. 3B, tailored arrows). Only 2 of 9 estradiol treated patients, one who received estradiol with antiandrogens over a period of 6 years and one who received estradiol only, showed single spermatids and a few spermatozoa (Fig. 3C, circle) in the seminiferous tubules. 2 patients, one taking estradiol with antiandrogen and the other with progestin, showed few spermatocytes but no spermatozoa in their tubules. 56% (5 out of 9) of the estradiol treated patients revealed only round cells with large cytoplasm and a central nucleus around the periphery of the tubules (Fig. 3A, arrows). Immunofluorescent staining for melanoma-associated antigen 4 (MAGEA4), a marker for spermatogonia, confirmed that these cells were spermatogonia (Fig. 3D, green staining). All patients displaying spermatogonia only within their seminiferous tubules received estradiol in combination with oral antiandrogens or gonadotropin-releasing hormone (GnRH) agonists.

### Effect of hormonal treatment on the morphology of Sertoli and Leydig cells

**Figure 4:**
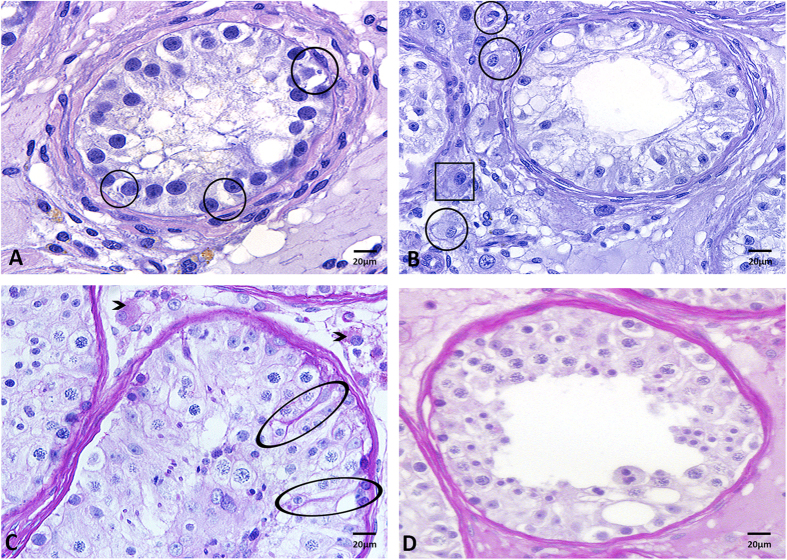
Effect of treatment with estradiol on Sertoli cell and Leydig cell morphology and glycoprotein expression. (**A**) Sertoli cell vacuolation was evident in all patients treated with estradiol (circle, H&E). (**B**) Following estradiol treatment, the majority of the Leydig cells appeared irregular in form and size and revealed signs of degeneration of the nucleus and cytoplasm (circle, H&E). Very few Leydig cells were only slightly altered (rectangle). (**C**) Glycoprotein synthesis was seen in the estradiol treated patients in both the cytoplasm of the Leydig cells (➤, PAS) and in the cytoplasm of some scattered Sertoli cells within the tubules (circle, PAS). (**D**) PAS staining with amylase confirmed that glycoproteins were synthesized in the estradiol treated patients.

All patients showed Sertoli cell vacuolation following treatment with estradiol (Fig. 4A, circles). In addition, most Leydig cells appeared fewer in number, irregularly shaped and with degradation of the nucleus and cytoplasm occurring (Fig. 4B, circles). Few Leydig cells revealing normal physiological morphology were detected (Fig. 4B, rectangle).

### Effect of hormonal treatment on glycoprotein production

Following estradiol treatment, all patients showed synthesis of glycoproteins both within the seminiferous tubules and in the connective tissue surrounding the tubules (Fig. 4C, pink staining). Within the tubules, Periodic acid-Schiff (PAS) staining was increased in the Sertoli cells (Fig. 4C, circle). The processes of the Sertoli cells surrounding the spermatogonia were distinctly stained (Fig. 4C, circle). Outside the tubules, large accumulations of glycoproteins were seen within the cytoplasm of Leydig cells (Fig. 4C, arrowheads) following estradiol treatment, which was not seen in patients without estradiol treatment. While some estradiol treated patients displayed glycogen within the tubules, this was not a notable amount nor consistent with any specific medications (Fig. 4D).

### Effect of hormonal treatment on Estrogen receptor alpha (ERα) expression

**Figure 5:**
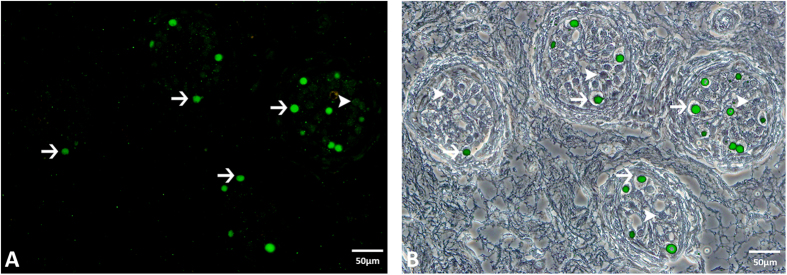
Effect of treatment with estradiol on ERα expression. (**A**) Estradiol treatment results in diminished expression of ERα in Sertoli cells, whereas expression in spermatogonia is not altered (→ spermatogonia, ➤ Sertoli cells). (**B**) Identical phase contrast image of (**A**) with fluorescent signal inserted demonstrating the spermatogonia staining positive for ERα and faint in Sertoli cells (→ spermatogonia, ➤ Sertoli cells).

In all estradiol treated patients, ERα was diminished in the Sertoli cells, independent of the treatment regimen (Fig. 5). Contrary to this, the spermatogonia, spermatocytes and spermatozoa (if present) showed a clear labelling which resembles the physiological expression as described in the literature^42^ (Fig. 5).

### Effect of different treatment regimens on the histomorphology of the testis

Interpretation of all histochemical data in correlation with treatment regimens revealed that the administration of estradiol in combination with antiandrogens or GnRH analogues always induced a complete arrest of spermatogenesis with spermatogonia only being present. The one patient who received estradiol in combination with the progestin dydrogesterone did additionally show some spermatocytes within the tubules. This was also the only patient who did not show fatty degeneration in the connective tissue. 2 patients had single spermatozoa in the tubules. One of these patients received estradiol only and the other received estradiol and antiandrogens for more than 6 years. Both of these patients also did not show an increase in thickness of the ECM and revealed the largest diameter of the seminiferous tubules, which resembled that of the patients without treatment. The effects observed on Sertoli cells, Leydig cells and on glycoprotein accumulation occurred in all patients irrespective of the treatment being estradiol only or in combination with antiandrogens or GnRH analogues. The one patient who received progesterone alongside estradiol and antiandrogens showed no difference in all histochemical and morphological parameters in comparison to the patients receiving estradiol and antiandrogens or GnRH agonists. The patient taking estradiol only had similar results as compared to the patient taking estradiol alongside antiandrogens for a period of 6 years in the histochemical parameters. Oral application of estradiol resulted in the same testicular alterations as those seen in the patients receiving local application of estradiol gels. Additional treatment with progesterone gel did not alter the effects seen. The effects of treatment were seen across all ages from 26 to 52. One patient was obese and had diabetes but this did not alter the results seen.

## Discussion

Our studies have shown that estradiol treatment exerts both inhibiting and stimulatory effects on testicular cells depending on the cell type it is acting on. The extent of the effects is modulated by estradiol as well as by the use of additional medications. In the testicular seminiferous tubules, spermatogenesis is distinctly impaired, resulting for the most part in complete azoospermia. According to our results, it is beneficial to apply estradiol in combination with antiandrogens or GnRH analogues to completely inhibit spermatogenesis. However, treatment with progestins or progesterone does not further increase the effects of estradiol and GnRH analogues or antiandrogens. On the other hand, it is important to know that estradiol treatment only, or treatment with estradiol and antiandrogens for a more extensive period than most, can result in spermatocytes, spermatids and even spermatozoa being present in the tubules. This is supported by the findings of Thiagaraj *et al*.^38^ who also described different levels of impaired spermatogenesis in transsexuals but did not correlate them to the medical record of the patients^38^. It is of interest that the patient receiving estradiol only displayed morphological features closest to the patient receiving estradiol and antiandrogens for the extensive period of 6 years. This suggests that long term administration of these drugs may result in decreased receptor responsiveness and a consequential increase in drug resistance. Another explanation might be an increased rate of elimination of the applied hormones within the body. We are the first to show that there is a significant decrease in the diameter of the seminiferous tubules in humans following estradiol treatment. This may explain the finding of Sapino *et al*.^37^ who described a 20–70% decrease in weight of the testes after estradiol treatment^37^. Interestingly, the 2 estradiol treated patients in our study displaying spermatids and spermatozoa, also had mean seminiferous tubule diameters most similar to that of the patients receiving no estradiol treatment. In light of the report by Gülkesen *et al*.^43^, who described a decrease in the diameter of the seminiferous tubules of hypospermatogenic men - a finding they noted had also been seen before in previous studies^44^,^45^, this suggests that a reduced tubule diameter is correlated with impaired spermatogenesis^43^. This finding is clinically important as it suggests that success rates of sperm retrieval during testicular sperm extraction (TESE) could be increased by selection of testicular tissue revealing the physiological range of diameter. Using MAGEA4 immunostaining we have shown that, in most patients, the only cells of spermatogenesis remaining in the tubules after estradiol treatment are spermatogonia. While estradiol has been shown to have negative impacts on the testis, it has also been shown to be a potent hormone supporting germ cell survival. This dual functionality has been highlighted in the literature^20^,^46^,^47^. In the patients investigated, the inhibitory effect of estradiol on spermatogenesis might be due to the estradiol induced impairment of Sertoli cell function. Impaired Sertoli cell function and disturbed communication with germ cells results in the induction of germ cell death^48^,^49^. This is supported by our results, which showed pronounced vacuolisation in the cytoplasm of the Sertoli cells after treatment with estradiol. Following the development of such vacuoles in Sertoli cells, immature germ cells are released from their bonds with Sertoli cells and prematurely transported to the epididymis^50^. It has been shown in murine Sertoli cells that estradiol works alongside follicle-stimulating hormone (FSH) during mRNA transcription of N-cadherin, a protein responsible for cell adhesion^21^,^32^. All of these findings imply that excess estradiol impairs the dynamic process of spermatogenesis driven by Sertoli cells separating and reforming tight junctions via N-cadherin. Glycoprotein accumulation occurred in Sertoli cells following estradiol treatment; this has a major impact on spermatogenesis because the glycoproteins in the Sertoli cells are pivotal for transport of ions and hormones, the remodelling processes during spermiation and the formation of structural components of the basement membrane^51^. Although the precise mechanisms are not fully elucidated yet, recent evidence suggests that the action of estradiol in Sertoli cell metabolism is mediated through modulation of glycolysis-related transporters and enzymes, particularly at the transcriptional level^52^. The action of estradiol is correlated to a functional ERα. As shown by our studies, the expression of the ERα is diminished in Sertoli cells. This might be due to overexposure to estradiol during the treatment or simply a result of the pronounced vacuolization of the cells negatively impacting the nuclear receptors.

Alongside alteration of Sertoli cell metabolism, estradiol treatment results in changes of the morphology of Leydig cells. Unlike their usual uniform shape and size, the Leydig cells of the estradiol treated patients appeared fewer in number and showed irregular form and size and signs of degeneration of the nucleus and cytoplasm. Leydig cells are responsible for production of testosterone which is essential for spermatogenesis to occur. It has been shown that excess estradiol exerts negative feedback on luteinizing hormone (LH), which in turn leads to reduced serum testosterone concentrations^53^. Lack of testosterone prevents germ cells from passing the meiotic stage of division^54^,^55^,^56^. As excess levels of estradiol inhibit FSH and LH secretion, both the Sertoli and Leydig cell functions are compromised which inevitably results in impaired fertility in the patient^57^.

Besides its distinct effects on the cells involved in spermatogenesis, treatment with estradiol also induces strong alterations in the connective tissue surrounding the seminiferous tubules. Considerable fatty degeneration occurred in the interstitial tissue surrounding the tubules after estradiol treatment. It has been shown that increased adipose tissue in obese individuals leads to marked elevation of extragonadal aromatase activity, which results in enhanced estradiol levels^58^. In addition to this, the hormone therapy of transsexuals involving additional administration of antiandrogens or GnRH agonists further decreases testosterone levels in the patient. Decreased testosterone levels again are known to be inversely associated with increased fat deposition and a consequent increase in estradiol production^59^. It is of special interest that the only patient who did not show fatty degeneration in the connective tissue took dydrogesterone, a strong acting progestin. Recently, it has been shown that several synthetic progestins are able to disrupt the aromatase expression in the zebra fish brain^60^. Thus, dydrogesterone might have blocked the increased aromatase activity caused by the elevated estradiol levels.

In addition to the occurrence of fatty degeneration, collagen synthesis within the ECM of the seminiferous tubules was increased after estradiol treatment. This is of particular interest because an increase in thickness of the ECM has been correlated with male infertility^61^. A correlation between hypospermatogenesis and ECM thickness has been demonstrated: testes from hypospermatogenic men showed an increase in thickness in the seminiferous tubule ECM alongside a decrease in diameter of the seminiferous tubules^43^. Similar effects are seen in the transsexual patients after estradiol treatment in this study, demonstrating a link between increased estradiol and enhanced synthesis of collagen fibers in the testicular tissue. Sertoli cells are involved in the deposition of ECM components such as collagen and laminin^62^,^63^. Thus, the increased collagen synthesis might also be a result of impaired Sertoli cell function.

In summary, our study has shown that treatment with estradiol results in severely impaired spermatogenesis which is correlated with increased synthesis of glycoproteins in Sertoli cells and Leydig cells and increased collagen synthesis and fatty degeneration in the testicular connective tissue. The results of this study have added new insights by quantifying the significant decrease in diameter of seminiferous tubules after hormonal treatment, by linking the different effects seen to different treatment regimens and by assessing the effects of estradiol treatment on ERα expression in the human testis. There is clear evidence from the data that treatment of patients with additional medications, such as androgen antagonists or GnRH agonists, alongside estradiol exacerbate the effects of estradiol treatment, particularly on spermatogenesis. This data might be of interest to doctors prescribing hormonal drugs to male-to-female transsexuals in order to optimise the treatment of each individual. Our results are also likely to provide novel concepts for treatment of males revealing excess estradiol levels. Male infertility is a condition attributed to hyperestrogenism and it has been postulated that it is more common than currently assumed^47^,^64^. Up to 15% of couples are suffering from infertility and 1 in 2 of these are a result of male infertility^65^,^66^,^67^,^68^. Thus, the results obtained in this study might contribute to developing new therapeutic strategies for male hyperestrogenism which focus on restoration of Sertoli cell and Leydig cell function and on management of dysregulations in glycoprotein and fat metabolism of the testicular connective tissue.

## Patients and Methods

### Ethical approval

The study was ethically approved by the Ethic Commission of the University of Munich (Nr. 161–12) and was performed according to the declaration of Helsinki.

### Informed consent

Informed and written consent was obtained from all patients before surgery.

### Patients

All patients were on estradiol treatment for 1–6 years prior to operation. The estradiol medication used by 8 patients was estradiol gel (Sisare^®^, Gynokadin Dosiergel^®^); the remaining patient was taking oral estradiol (Estrofem^®^). 8 patients received additional medication in form of a) GnRH analogues (goserelin acetate, Zoladex^®^), b) antiandrogens (cyproterone acetate, Androcur^®^), c) progestins (Duphaston^®^) or d) progesterone (Progestogel^®^). Treatment regimen, dose and duration of treatment are outlined in Table 1. Testicular tissue was obtained from all 9 male-to-female transsexual patients (aged 26–52) immediately after orchiectomy. All tissues were immediately fixed in Bouin’s fluid, embedded and sectioned. For comparative analyses tissue samples were taken from the microscopically unaltered testicular tissues surrounding tumors from 7 testes tumor patients immediately after tumor removal. None of these patients were on any medication prior to surgery. As 3 patients were shown to be affected in the tissue surrounding the tumors following hematoxylin and eosin (H&E) staining, they were excluded from the study. Unaltered testicular tissue surrounding tumours were the only suitable controls as testicular samples obtained from forensic medicine were degenerated by the time legal permission was given.

### Stereomicroscopy

The diameters of the seminiferous tubules were measured at x100 magnification on the sections from the estradiol treated transsexual patients (9) and the sections from patients without estradiol treatment (4) using CellSens Standard (Olympus) image processing software. Only circular tubules were measured on each patient’s section and the entire slide was assessed (on average 144 tubules were measured per slide). Two slides were analysed per patient.

### Morphological analysis

Sections were fixed in Bouin’s fluid, dehydrated in graded dilutions of ethanols (70–100%) and embedded in paraffin. For staining, 3 μm sections were cut and mounted on Superfrost Plus slides (Thermo Scientific, Dublin, Ireland). Cryosections were prepared as follows: testicular tissue samples (1 cm) were snap-frozen in liquid nitrogen immediately after removal. After cryotomy, sections (8 μm) were put on coated slides (Superfrost Plus, Menzel, Braunschweig, Germany) and dried at room temperature. The slides were frozen at −20 °C until use.

Hematoxylin and Eosin (H&E) staining was carried out to investigate testicular morphology. Following dewaxing in xylene (Sigma-Aldrich, Wicklow, Ireland; room temperature [RT], 3 × 10 minutes [min]), rehydration in descending alcohols (RT, 100% 5 min, 100% 5 min, 80% 5 min, 70% 5 min) and distilled water (RT, 5 min), sections were put in hematoxylin (VWR, Dublin, Ireland; RT, 30 seconds [s]), running water (15 min), eosin (Sigma-Aldrich, Wicklow, Ireland; RT, 45 s) and then running water (5 min). Sections were then dehydrated in ascending alcohol solutions (RT, 70% 5 min, 80% 5 min, 100% 5 min, 100% 5 min), cleared in xylene (Sigma-Aldrich, Wicklow, Ireland; RT, 3 × 10 min), and mounted in DPX (Sigma-Aldrich, Wicklow, Ireland).

### Histochemistry

Periodic acid-Schiff (PAS) staining was carried out with and without amylase digestion for detecting glycogen and glycoproteins, respectively. Following dewaxing in xylene (Sigma-Aldrich, Wicklow, Ireland; RT, 3 × 10 min), rehydration in descending alcohols (RT, 100–70%) and distilled water (RT, 5 min), sections were put in 0.25% periodic acid (Sigma-Aldrich, Wicklow, Ireland; RT, 3.5 min), running water (10 min), distilled water (rinsed briefly, RT), Schiff’s reagent (Sigma-Aldrich, Wicklow, Ireland; RT, 5 min), sulfite water (0.5 g sodium disulfate, 99 ml distilled water, 1 ml 25% hydrochloric acid [HCL]. Sigma-Aldrich, Wicklow, Ireland; RT, 2 × 5 min), running water (10 min), hematoxylin (counterstain, VWR, Dublin, Ireland; RT, 30 sec), and running water (10 min). Sections were then dehydrated in ascending alcohol solutions (70–100%), cleared in xylene (Sigma-Aldrich, Wicklow, Ireland; RT, 3 × 10 min), and mounted in DPX (Sigma-Aldrich, Wicklow, Ireland). The same protocol was carried out with slides pretreated with amylase (Sigma-Aldrich, Wicklow, Ireland; 37 °C, 10 min) before incubation in periodic acid. Amylase digests glycogen so that glycogen and glycoproteins can be differentiated.

Picrosirius red staining was carried out for localization of collagen around the tubules. Sections were dewaxed in xylene (Sigma-Aldrich, Wicklow, Ireland; RT, 3 × 10 min), rehydrated in descending alcohols (RT, 100–70%) and put in picrosirius red/fast green solution in the dark (0.1% direct red 80 solution and 0.1% fast green solution, made up in aqueous picric acid solution at a ratio of 1:9 dye: picric acid solution. Sigma-Aldrich, Wicklow, Ireland; RT, 2 hours [hr]). They were placed in distilled water (RT, 2 × 2 min), dehydrated in a series of ascending alcohol solutions (RT, 70–100%), cleared in xylene (Sigma-Aldrich, Wicklow, Ireland; RT, 3 × 10 min), and mounted in DPX (Sigma-Aldrich, Wicklow, Ireland).

Sudan Red staining was carried out as follows:

For localization of fat vacuoles in tissue outside of the tubules, cryosections were thawed, washed twice in phosphate buffered saline (PBS) (RT, 5 min), pre-treated with 60% isopropylene (Merck, Germany, RT, 5 min), and finally put in oil red (Merck, Germany; RT, 10 min). Following treatment the specimens were washed in distilled water and counterstained with hematoxylin (Merck, Germany).

### Immunohistochemistry

#### Immunohistochemistry using MAGEA4

Paraffin sections were dewaxed in xylene (Sigma-Aldrich, Wicklow, Ireland; RT, 3 × 10 min) and rehydrated in descending alcohols (100–70%). Antigen retrieval was carried out using citrate acid buffer in a high pressure cooker (pH 6, 20 min). Slides were then permeabilized in 10% Triton X-100 (VWR, Dublin, Ireland; RT, 10 min), rinsed with PBS-Tween (Sigma-Aldrich, Wicklow, Ireland) and blocked in 10% goat serum (Sigma-Aldrich, Wicklow, Ireland; RT, 1hr). The slides were then incubated with a MAGEA4 primary antibody (1:100 in PBS-Tween, 4 °C, overnight). Anti-MAGEA4 is a mouse monoclonal antibody purified from hybridoma 57B, and enables differentiation between spermatogonia and Sertoli cells^69^. Following incubation, the slides were washed in PBS-Tween (Sigma-Aldrich, Wicklow, Ireland; RT, 3 × 5 min), incubated in Alexa Fluor-488 secondary antibody (Bio-Sciences, Dublin, Ireland; RT, 1 hr, 1:500) in the dark, washed in PBS-Tween (Sigma-Aldrich, Wicklow, Ireland; RT, 3 × 5 min), mounted with Fluoromount (Sigma-Aldrich, Wicklow, Ireland), and coverslipped. Microscopy was performed using the Olympus BX51 microscope and an Olympus DP71 camera (Masontec, Dublin, Ireland).

### Immunohistochemistry for localization of ERα

Paraffin sections were dewaxed in xylene (Sigma-Aldrich, Wicklow, Ireland; RT; 3 × 10 min) and rehydrated in descending alcohols (100–70%). Sections were rinsed in PBS (Thermo Scientific, Dublin, Ireland; 5 min). Antigen retrieval was carried out using citrate acid buffer in a microwave oven (pH 6, 5 mins). Slides were left at RT for 8 min and washed in PBS (Thermo Scientific, Dublin, Ireland; 3 × 5 min). Slides were placed in bovine serum albumin (BSA, Sigma-Aldrich, Wicklow, Ireland; 10 min) and washed in PBS (Thermo Scientific, Dublin, Ireland; 3 × 5 min). Slides were blocked in 10% normal goat serum (Sigma-Aldrich, Wicklow, Ireland; RT, 1 hr) and incubated in the primary antibody [anti-ERα rabbit polyclonal antibody (MC-20: sc-542, Santa Cruz Biotechnology, Santa Cruz, USA) 1:50 in PBS, 4 °C, overnight]. The slides were washed in PBS (Thermo Scientific, Dublin, Ireland; 3 × 5 min) and incubated in Alexa Fluor 488 labelled secondary antibody [goat anti-Rabbit IgG (H + L), Alexa Fluor^®^ 488 conjugate; Thermo Scientific, Dublin, Ireland; dilution 1:500, 1 hr]. After incubation, the slides were washed in PBS (Thermo Scientific, Dublin, Ireland; 3 × 5 min), mounted with Fluoromount (Sigma-Aldrich, Wicklow, Ireland) and coverslipped. Microscopy was performed using the Olympus BX51 microscope and an Olympus DP71 camera (Masontec, Dublin, Ireland). In order to be able to compare immunohistochemical staining and morphological pictures, phase contrast microscopy (Olympus BX51 microscope) was applied on the sections after fluorescent imaging. The pictures were merged using the software Adobe Photoshop.

### Scanning Electron Microscopy

After removal, the tissues were washed in Soerensen’s buffer twice (pH 7.4, 1:5 solution of 0.07 M KH_2_PO_4_, and 0.07 M Na_2_HPO_4_-2H_2_O) and fixed in 1% glutaraldehyde in Soerensen’s buffer (4 °C, 24 hr). Tissues were washed again in Soerensen’s buffer and dehydrated in a series of ascending acetones (RT; 10%, 20%, 30%, 40%, 50% and 60% x2, 5 min; 70%, 80% and 90%, 1 hr, 100%, 12 hr). A union point dryer CPD 030 (Bal-Tec, Walluf, Germany) was used to dry the tissue samples. For visualisation of the lumina of the seminiferous tubules, the samples were dipped in liquid nitrogen and mechanically broken into several pieces using a forceps. The pieces were coated with 12-nm gold-palladium using the Union SCD 040 sputtering device (Bal- Tec, Walluf, Germany). Analyses were performed with a Zeiss scanning electron microscope DSM 950 at magnifications of x200 to x15,000.

### Statistical analyses

The diameter of the seminiferous tubules in the estradiol treated patients and patients without estradiol treatment were compared using a 2-sided *t*-test using SPSS software. P < 0.05 was considered significant.

## Additional Information

**How to cite this article**: Leavy, M. *et al*. Effects of Elevated β-Estradiol Levels on the Functional Morphology of the Testis - New Insights. *Sci. Rep.* **7**, 39931; doi: 10.1038/srep39931 (2017).

**Publisher's note:** Springer Nature remains neutral with regard to jurisdictional claims in published maps and institutional affiliations.

## Figures and Tables

**Table 1:** Type of hormonal treatment, dosage and duration of treatment.

Patient number	Age	Treatment Regimen
1	26	Estradiol gel (Sisare^®^). 2 mg/day for 21 months. GnRH analog: goserelin acetate (Zoladex^®^). 10.8 mg implant 3 times a month as start up, then every 3 months. 21 months in total.
2	49	Estradiol gel (Gynokadin Dosiergel^®^). 4 mg/day as start up, then 2 mg/day. 6 years in total. Antiandrogen: cyproterone acetate (Androcur^®^). 10 mg/day for 6 years.
3	47	Oral Estradiol (Estrofem^®^). 2 mg/day for 20 months. Antiandrogen: cyproterone acetate (Androcur^®^). 10 mg/day for 20 months.
4	52	Estradiol gel (Sisare^®^). 2 mg/day for 24 months. Progestin: Dydrogesterone (Duphaston^®^). 10 mg/day for 24 months.
5	40	Estradiol gel (Gynokadin Dosiergel^®^). 2 mg/day for 24 months. Weaned 9 days before surgery. Antiandrogen: cyproterone acetate (Androcur^®^). 10 mg/day for 24 months. Progesterone gel (Progestogel^®^) 2 mg/day for 24 months. Weaned 9 days before surgery.
6	26	Estradiol gel (Gynokadin Dosiergel^®^) 2 mg/day for 24 months.
7	48	Estradiol gel (Sisare^®^). 2 mg/day for 36 months. Antiandrogen: cyproterone acetate (Androcur^®^). 10 mg/day for 36 months.
8	41	Estradiol gel (Gynokadin Dosiergel^®^). 2 mg/day for 18 months. Antiandrogen: cyproterone acetate (Androcur^®^). 10 mg/day for 18 months.
9	46	Estradiol gel (Sisare^®^). 4 mg/day for 36 months. Antiandrogen: cyproterone acetate (Androcur^®^). 15 mg/day for 36 months.
